# Kornblith versus Sosa on grades of knowledge

**DOI:** 10.1007/s11229-018-1689-8

**Published:** 2018-01-16

**Authors:** J. Adam Carter, Robin McKenna

**Affiliations:** 1 Department of Philosophy, University of Glasgow, 67 Oakfield Avenue, Glasgow, G12 8LP Scotland, UK; 2 Institut für Philosophie, University of Vienna, Universitätsstrasse 7 (NIG), 1010 Vienna, Austria

**Keywords:** Ernest Sosa, Virtue epistemology, Animal knowledge, Reflective knowledge, Analysis of knowledge

## Abstract

In a series of works Sosa (in: Knowledge in perspective, Cambridge University Press, Cambridge, 1991; A virtue epistemology: apt belief and reflective knowledge, Oxford University Press, Oxford, 2007; Reflective knowledge: apt belief and reflective knowledge, Oxford University Press, Oxford, 2009; ‘How Competence Matters in Epistemology’, Philos Perspect 24(1):465–475, 2010; Knowing full well, Princeton University Press, Princeton, 2011; Judgment and agency, Oxford University Press, Oxford, 2015; Epistemology, Princeton University Press, Princeton, 2017) has defended the view that there are two kinds or ‘grades’ of knowledge, animal and reflective. One of the most persistent critics of Sosa’s attempts to bifurcate knowledge is Kornblith (in: Greco (ed) Ernest sosa and his critics, Wiley, Hoboken, 2004; ‘Sosa in Perspective’, Philos Stud 144(1):127–136, 2009; On reflection, Oxford University Press, Oxford, 2012). Our aim in this paper is to outline and evaluate Kornblith’s criticisms. We will argue that, while they raise a range of difficult (exegetical and substantive) questions about Sosa’s ‘bi-level’ epistemology, Sosa has the resources to adequately respond to all of them. Thus, this paper is a (qualified) defence of Sosa’s bi-level epistemology.

## Introductory remarks

In a series of works Ernest Sosa (see Sosa 1991, 2007, 2009, 2010, 2011, 2015, 2017) has defended the view that there are two kinds or ‘grades’ of knowledge, animal and reflective. Put roughly, Sosa holds that one has animal knowledge that p iff one truly believes that p as the result of one’s exercising a reliable competence, whereas one has reflective knowledge that p iff one has (a) animal knowledge that p; and (b) achieved a perspective on one’s first-order belief from which one can see it as reliably produced and fitting into a broader network of first-order beliefs. One of the most persistent critics of Sosa’s attempts to bifurcate knowledge is Hilary Kornblith (see Kornblith 2004, 2009, 2012). Our aim in this paper is to outline and evaluate Kornblith’s criticisms. We will argue that, while they raise a range of difficult (exegetical and substantive) questions about Sosa’s ‘bi-level’ epistemology, Sosa has the resources to adequately respond to all of them. Thus, this paper is a (qualified) defence of Sosa’s bi-level epistemology.

## Sosa on grades of knowledge

In this section we will start by outlining the contours of Sosa’s reflective/animal knowledge distinction. We will then articulate three conditions that Sosa’s distinction must meet if it is to be adequate.

While Sosa’s distinction has retained the same broad shape over the years, the details have varied in interesting ways. We will highlight three ‘stages’ in its development, which we draw on in the rest of our discussion. As we will see in §4, the most recent stage represents a significant improvement on the previous versions. In the first stage (see Sosa 1991), Sosa puts the distinction like this:

One has *animal knowledge* about one’s environment, one’s past, and one’s own experience if one’s judgments and beliefs about these are direct responses to their impact—e.g. through perception or memory—with little or no benefit of reflection or understanding.

One has *reflective knowledge *if one’s judgment or belief manifests not only such direct response to the fact known but also understanding of its place in a wider whole that includes one’s belief and knowledge of it and how these come about (1991, p. 240).

One has animal knowledge that p iff one has a competently produced true belief that p. Reflective knowledge differs from animal knowledge in that it requires an understanding of the process that produced one’s belief, and of how it fits into a broader network of beliefs. Note that by “understanding” Sosa means more *animal *knowledge.^1^ One has reflective knowledge that p iff one has (a) animal knowledge that p, and, (b) animal knowledge of how one’s belief came about and of how it fits into a broader network of beliefs.

In the second stage (see Sosa 2007, 2009), Sosa introduced some now-familiar terminology:

We can distinguish between a belief’s accuracy, i.e., its truth; its adroitness, i.e., its manifesting epistemic virtue or competence; and its aptness, i.e., its being true *because *competent. Animal knowledge is essentially apt belief (2007, pp. 23–24).

Reflective knowledge goes beyond animal knowledge, and requires also an apt apprehension that the object-level perceptual belief is apt. What competence might a believer exercise in gaining such meta-apprehension? It would have to be a competence enabling him to size up the appropriateness of the conditions (2007, p. 108).

There are important differences between this account and Sosa’s earlier account. Sosa thinks of beliefs as a species of performance,^2^ and any performance with an aim can be evaluated along three dimensions:

(i) whether it is *accurate* (whether it attains its aim),
(ii) whether it is *adroit* (whether it manifests competence),
(iii) whether the success is accurate because adroit (whether it is *apt*).

Diana, a highly skilled archer, fires a shot at a target. The shot is accurate if it hits the target, adroit if it manifests Diana’s archery competence, and apt if it hits the target because it manifests Diana’s archery competence, rather than because (say) a gust of wind blew it off course before another gust blew it back on course. Similarly, a belief is apt iff it achieves its aim (truth) because of an exercise of an epistemic competence. Sosa’s account of aptness builds in that the success (achieving the aim) must be sufficiently attributable to the exercise of the relevant competence. These differences apply at the level of animal and reflective knowledge. Thus, one has animal knowledge (that p) iff one has a true belief (that p) that is sufficiently creditable to the relevant epistemic competence, and one has reflective knowledge (that p) iff one has (a) animal knowledge (that p), and, (b) true beliefs about how one’s belief that p came about (and about how it fits into a broader network of beliefs) that themselves are sufficiently creditable to a ‘meta-competence’ (a competence in evaluating one’s first-order competences).

In the third stage (see Sosa 2010, 2011, 2015), Sosa introduces the idea of *knowing full well*. The picture at the level of animal knowledge is much the same. The crucial difference is at the level of reflective knowledge (or ‘knowing full well’).^3^ While the view is complex, the basic idea is that reflective knowledge (that p) is not just the conjunction of apt and meta-apt belief (that p). Rather, one has reflective knowledge only if one’s apt belief is *guided *by one’s meta-apt belief.

Because it is important to understand this idea of guidance for what follows, we will give a quick explanation of it. Consider performances in general. Diana takes an archery shot and hits the selected target. We can evaluate her archery performance on two levels. On the first level, we can evaluate it for aptness. Her shot was apt iff it hit the target in a way that is sufficiently creditable to her archery competence. On the second level, we can evaluate it in terms of whether the shot’s aptness is sufficiently creditable to a ‘meta-competence’ in selecting when to shoot. Sosa’s idea is that her shot was successful on this level iff this meta-competence (rather than, say, a coin-flip or some other unreliable process for selecting shots) guided her selection of the shot and the broader process which led to its subsequent success. Similarly, we can evaluate beliefs on two levels. Helen stands in front of a tree and forms the belief that there is a tree. On the first level, we can evaluate her belief for aptness. The belief is apt iff its truth is sufficiently creditable to the relevant epistemic competence. On the second level, we can evaluate it in terms of whether its aptness is sufficiently creditable to a ‘meta-competence’ in evaluating her first-order epistemic competences. Again, Sosa’s idea is that her belief was successful on this level iff this meta-competence guided her forming of the first-order belief and the broader process that led to its subsequent success.^4^

In summary: Sosa holds that animal knowledge (that p) is just apt belief (that p). What reflective knowledge requires has changed over the years. In his most recent work, reflective knowledge is animal knowledge (apt belief) that is guided by a meta-competence, that is, a competence in evaluating the likelihood of success via one’s first-order competences. When one has apt belief that is guided in this way, one knows full well.

Turning now to the adequacy conditions, we think that Sosa’s reflective/animal knowledge distinction must do three things:

*Epistemological adequacy*: It must be part of a plausible more general epistemological picture.

*Non-triviality*: It must not boil down to a familiar distinction, such as the distinction between first-order knowledge (knowing that p) and second-order knowledge (knowing that you know that p).

*Non-arbitrariness*: It must be worth drawing.

We will briefly motivate each condition. First, some of Sosa’s formulations give the impression that the distinction between animal and reflective knowledge is similar to (or even identical with) the distinction between first- and second-order knowledge. But we hardly need to invoke a distinction between reflective and animal knowledge here, because we already have a perfectly good distinction between first-order and second-order knowledge—one that has been extensively discussed in epistemology (including formal epistemology) and in game theory.^5^ So, impressions notwithstanding, Sosa’s distinction can’t just boil down to the distinction between first- and second-order knowledge.

Second, there are vast *bodies* of knowledge that humans can have which animals can’t. Consider scientific knowledge, higher mathematics, or knowledge about the epistemic status of our beliefs (we are assuming that animals are incapable of forming second-order mental states). But we don’t need to invoke a distinction between human and animal knowledge to explain the fact that animals lack scientific or mathematical knowledge, or can’t have second-order knowledge.

Finally, as for *non-arbitrariness*, while it is hard to say anything precise about when a distinction is worth drawing, it is clearly important that the reflective/animal knowledge distinction be worth drawing. Consider some distinctions that don’t seem worth drawing:

(i) Knowledge obtained on weekdays versus knowledge obtained on the weekend.
(ii) Knowledge obtained in Europe versus knowledge obtained outside of Europe.
(iii) Knowledge about redness versus knowledge not about redness.
(iv) Knowledge obtained by people with low IQs versus knowledge attained by people with high IQs

There are conceivable theoretical purposes that could lead one to make these distinctions, but to show that one’s theoretical purposes require them would be a tough task, and one can always question the value of the theoretical purpose.

We are going to argue that Sosa’s reflective/animal knowledge distinction satisfies all three conditions. We start with the first condition.

## The first condition: fake barn Cases

The first adequacy condition is that Sosa’s distinction must be part of a plausible more general epistemological picture. Evaluating the plausibility of Sosa’s epistemological picture is an extensive task, and we can’t speak to all aspects of his view here. We focus on an objection from Kornblith (2009) which, if sound, spells serious trouble for Sosa. Put briefly, Sosa holds that subjects have animal knowledge in fake barn cases, but denies that they have reflective knowledge. Thus, he thinks his account can ‘split the difference’ between any inclination we have to say that subjects in fake barn cases know and the inclination we have to deny that they know.^6^ Kornblith’s complaint is that this asymmetric treatment of fake barn cases is unmotivated. Sosa has to either accept that there isn’t animal knowledge in fake barn cases, or accept that there is both animal and reflective knowledge. We think that both horns of this dilemma are unpalatable. But we are going to argue that the dilemma doesn’t arise in the first place because Sosa’s asymmetric treatment of fake barn cases can be motivated. Thus, Kornblith’s objection fails.

We can start with Sosa’s treatment of fake barn cases. Consider a standard fake barn case:

fakebarns: Using his reliable perceptual faculties, Barney non-inferentially forms a true belief that the object in front of him is a barn. Barney is indeed looking at a barn. Unbeknownst to Barney, however, he is in an epistemically unfriendly environment when it comes to making observations of this sort, since most objects that look like barns in these parts are in fact barn façades.^7^

Whether Barney has animal or reflective knowledge depends on whether the relevant beliefs are (a) true (b) manifest the relevant (meta-)competences (c) their truth is sufficiently creditable to these (meta-)competences. On Sosa’s view, competences are *dispositions*, and dispositions are comprised of three aspects: seat, shape and situation. Here’s Sosa explaining these three components with reference to driving competence:

With regard to driving competence (or ability), for example, we can distinguish between (a) the innermost driving competence: that is, the structural seat in one’s brain, nervous system, and body, which the driver retains even while asleep or drunk, (b) the fuller inner competence, which requires also that one be in proper shape, i.e., awake, sober, alert, etc., and (c) the complete competence or ability to drive well and safely, which requires also that one be situated with control of a vehicle, along with appropriate road conditions pertaining to the surface, the lighting, etc. The complete competence is thus an SSS (or a SeShSi) competence (2015, p. 96).

Sosa allows that the shape or situation components may hold even though they *could easily have not held*. One can manifest one’s driving competence even though one could easily have been drunk, or could easily have been sitting in a broken car. The ‘modal fragility’ of one’s shape and situation need not prevent one from manifesting the relevant competence provided one actually *is* in good enough shape and appropriately situated.

This furnishes a simple explanation why Barney has animal knowledge. His belief is (a) true, (b) manifests the relevant perceptual competence and (c) its truth is sufficiently creditable to this perceptual competence. Of course, Barney’s belief could easily have been false; he could easily have been standing in front of a fake barn. But, because the modal fragility of his situation need not prevent him from manifesting the relevant competence, the fact that he could very easily have been in a situation where he would not have been competent to spot a barn is irrelevant.

What about reflective knowledge? Sosa says this about a structurally similar case, where the ‘kaleidoscope perceiver’ believes the wall in front of them is red, and it is indeed red, but unbeknownst to him a ‘jokester’ is controlling both the colour of the wall and the lighting conditions, and so could easily have made it such that the wall was white but bathed in red light:

If the kaleidoscope perceiver’s meta-competence is to yield knowledge, therefore, it must not be excessively liable to yield a falsehood when exercised in its appropriate conditions. Given the jokester, however, this requirement is not met, since too easily then might the perceiver have been misled in trusting the conditions to be appropriate in that default way. The kaleidoscope perceiver has animal knowledge but lacks reflective knowledge. He has apt belief simpliciter, but lacks apt belief aptly presumed to be apt (2007, p. 109).

This suggests that one has reflective knowledge (that p) only if the relevant meta-competence is not excessively likely to yield a false belief (about whether one has animal knowledge that p). At first sight, this is hard to square with what Sosa says about competences in general. As Kornblith (2009, pp. 131–33) points out, one would think that, if the modal fragility of Barney’s perceptual competence doesn’t interfere with the aptness of his first-order belief, the modal fragility of Barney’s meta-competence can’t interfere with the meta-aptness of his second-order belief. That is, one would think that reasons for holding that the situation component of the first-order competence holds are also reasons for thinking that the situation component of the meta-competence holds. But, as we will argue, there is a principled reason for this differential treatment.

First, why think that the situation component holds at the first order? Sosa invites us to consider three cases that, in his view, stand or fall together. The first case is Barney; the second is Kyle (the hero of the kaleidoscope perceiver case, explained above); the third is Simone, whose situation he describes as follows:

Simone [...] nears the end of her pilot training and each morning might, to all appearances, from her viewpoint, as easily be piloting in a simulator as in a real plane. When she happens to be really in flight, as she believes, can she know that she is? How could she, given that she might just as easily be in a simulator, with no way to tell the difference? (2017, p. 88).

According to Sosa, Barney, Kyle and Simone are to be treated symmetrically because:

(i) all retain their relevant inner competence or skill (i.e., the seat of their competences)
(ii) all are in good shape to exercise that competence
(iii) each lacks (or is in danger of lacking) the situation required for the complete competence (Kyle might easily have seen the surface in red light, Simone might easily have been in the simulation and Barney might easily have seen a fake barn).

If we assume that all three cases should be diagnosed symmetrically, then we have two options. We can deny that any of these performances are apt, or we can hold that they are all apt. Sosa, in evaluating the first option, argues that it is intractably problematic, as it would commit us to a double standard in our evaluations. This is because:

we impose no such requirement on archers, pilot trainees, or athletes. A basketball player, for example, might be in an indoor venue where his shots are calmly apt, even though high winds would impair them in all nearby venues where he might easily have been shooting (2010, p. 469).

Sosa’s argument is that, if we want to hold that Barney, Kyle and Simone fail to have apt beliefs—beliefs that are not attributable to the relevant first-order competences—because of the modal closeness of error then, by parity of reasoning, we need to hold that the basketball player who might easily have been in a windy venue fails to shoot aptly. Because it is counterintuitive to deny that the basketball player’s shots are apt, we shouldn’t deny that Barney’s, Kyle’s and Simone’s beliefs are apt.

Second, if one grants Sosa’s assumption that all these cases need to be treated symmetrically, and so that the situation component holds at the first order, why then deny that it holds at the second order? This requires appreciating why a first-order competence can be present even when the relevant second-order competence is absent. This can be the case, Sosa says, if the subject is ‘ill-placed to determine its presence’, where the subject:

is ill-placed, even with the first-order competence present, *so long as there would be no signs of its absence*. It does not matter whether the competence is there or not on the first order. What matters is that the subject is unable to discern its presence or absence. Because this deprives him of the required competence on the second-order, he is inappropriately situated (2010, pp. 473–74).

So Barney, Kyle and Simone are ill-placed to determine whether their first-order competences are present because they are unable to discern their presence. The thought, then, is that exercising a first-order competence does not require an ability to discern its presence (or absence), whereas exercising a second-order competence requires an ability to discern the presence (or absence) of relevant first-order competences. While Sosa doesn’t quite put it this way, we take this to fall out of the nature of second-order competences. A second-order competence is a competence in evaluating first-order competences, so exercising it requires a kind of sensitivity to whether one is in an appropriate situation for the exercise of one’s first-order competence. Because first-order competences aren’t competences in evaluating other competences, there is no equivalent requirement on them.

Putting this all together, Barney has animal knowledge because he is in a situation in which he can exercise his perceptual competence, even though he could easily not have been. Barney lacks reflective knowledge because he is not in a situation in which he can exercise the relevant meta-competence. He is not in such a situation because he is unable to discern the presence of the relevant perceptual competence. While this account raises several questions, the asymmetrical treatment of animal and reflective knowledge is well-motivated within Sosa’s framework. Thus, we—provisionally—conclude that Sosa’s account is epistemologically adequate.

## The second condition: the KK principle

The second adequacy condition is that Sosa’s distinction can’t just boil down to the familiar distinction between knowing that p (animal knowledge) and knowing that one knows that p (reflective knowledge). Here is Kornblith arguing that this adequacy condition is not met:

Sosa now defines animal knowledge as apt belief (roughly, reliably produced true belief), and reflective knowledge as ‘apt belief aptly noted’, i.e. true belief which is both reliably produced and also such that one has a reliably produced true belief that the first-order belief was reliably produced [see Sosa (2007, pp. 34, 43, 98, 113)]. But this new definition of reflective knowledge is just animal knowledge twice over. It requires no knowledge (reflective or otherwise) of how one’s first-order belief came about or its relationship to one’s other beliefs (Kornblith 2012, p. 15).

This worry is not without ground. Sosa sometimes says things like this:

If K represents animal knowledge and K+ reflective knowledge, then the basic idea may be represented thus: K+p $\leftrightarrow$ KKp (2007, p. 32).

But, while some of the things that Sosa says may give the impression that reflective knowledge is just animal knowledge “twice over”, this impression is, at best, misleading. To start, it is important to note that Sosa’s considered view is not (and never has been) that one has reflective knowledge that p if one has animal knowledge that one has animal knowledge that p. One requires, in addition, *more *animal knowledge, for instance, about how the relevant first-order belief came about, or how it fits into a broader network of beliefs.

One might respond on behalf of Kornblith that, even if this is Sosa’s considered view, there is still a sense in which reflective knowledge is just animal knowledge “twice over”. It may be that reflective knowledge that p isn’t just animal knowledge that one has animal knowledge that p. But it is just *more *animal knowledge. To sharpen this worry, compare Sosa’s distinction with Russell’s (1910) distinction between knowledge by acquaintance and knowledge by description. For Russell, this is a distinction between two different *epistemological kinds*. Knowledge by acquaintance is unmediated—as Russell puts it, it is a ‘direct cognitive relation’ (1910, p. 108)—whereas knowledge by description involves at least one inferential step.^8^ So the worry is that animal and reflective knowledge are of the same epistemological kind.

While this may be a problem for the earlier versions of the reflective/animal knowledge distinction, the most recent development in Sosa’s view outlined in §1 block it. What Sosa calls ‘knowing full well’ is animal knowledge that is *guided *by further animal knowledge. It isn’t enough to know full well (that p) that one has animal knowledge that p plus animal knowledge about the reliability of the relevant first-order competences (or, for that matter, any other animal knowledge about the relevant first-order competences). Rather, this extra animal knowledge must guide the process by which one arrived at the relevant first-order beliefs. Sosa’s distinction between animal knowledge and knowing full well therefore marks a distinction between two different epistemological kinds. Animal knowledge is a sort of direct response to the facts that is neither mediated nor guided by any competence in evaluating one’s first-order competences. Reflective knowledge is a response to the facts that is guided by these further competences. We conclude that, some misleading formulations notwithstanding, Sosa’s picture satisfies the second adequacy condition.

## The third condition: competences, value and anti-sceptical import

The third and final adequacy condition is that the distinction must be *worth drawing*. In this section we look at three ways in which Sosa might argue that his distinction is worth drawing:

(i) By appealing to the different *competences *involved in animal and reflective knowledge
(ii) By appealing to a difference in *value *between animal and reflective knowledge
(iii) By appealing to the special role of reflective knowledge in a viable anti-sceptical project.

We argue that, while (i) fails, both (ii) and (iii) can be defended. Thus, Sosa’s account satisfies the final adequacy condition.^9^

*First Way: Different Competences*

One might argue that the distinction between reflective and animal knowledge is worth drawing because they involve different skills (or, as Sosa would say, competences). Here is Timothy Perrine making this suggestion on behalf of Sosa:

For Sosa, knowledge is a kind of excellence [sic.] performance of a cognizer. Thus, for there to be two different kinds of knowledge, there would have to be two different kinds of skills that a cognizer can perform. But animal knowledge and reflective knowledge involve two different general kinds of skills that a cognizer can perform. (At the very least, Kornblith has given us no reason for thinking that they are not). So, Kornblith’s challenge can be met: there is a reason for thinking that Sosa has identified *two different kinds of knowledge *(Perrine 2014, p. 354)

The thought is that animal knowledge requires skilful use of one’s perceptual and other basic faculties, whereas reflective knowledge requires skilful reflection.^10^

The problem with this suggestion is that it faces a proliferation problem. If one can base a distinction between kinds of knowledge on a distinction between kinds of skills, one is going to have to countenance kinds of knowledge other than animal or reflective knowledge. Here is Kornblith making this point:

[S]o-called consultative knowledge is not a different kind of knowledge from non-consultative knowledge. Yes, in order to possess consultative knowledge, one must have gone through a certain process which the possessor of non-consultative knowledge has not followed. But if this is a ground for drawing a distinction between different kinds of knowledge, then we will have as many different kinds of knowledge as there are processes of belief acquisition and retention. Surely this multiplies kinds of knowledge far beyond necessity (2012, p. 19).

Kornblith does over-state the point here. Perrine’s claim is that there is a different kind of knowledge for every kind of *skill*, not that there is a different kind for every process of belief acquisition and retention. But, if reflective knowledge differs from animal knowledge because it requires skilful reflection rather than skilful use of one’s perceptual faculties, then what Kornblith calls consultative knowledge—knowledge one gets from consulting with others—differs from animal knowledge because it requires skilful consultation. Of course, one could conclude that there are three kinds of knowledge: animal, reflective and consultative. But the simpler conclusion is that you can’t base a distinction in kinds of knowledge on the fact that knowing can require the exercise of a range of different skills.

*Second Way: Difference in Value*

One might instead argue that reflective knowledge differs from animal knowledge in that it is either more valuable than animal knowledge, or valuable in a different way to animal knowledge. Sosa suggests this in several places. For instance:

Since a direct response supplemented by such understanding would in general have a better chance of being right, reflective knowledge is better justified than corresponding animal knowledge (1991, p. 240).

What favors reflective over unreflective knowledge? Reflective acquisition of knowledge is, again, like attaining a prized objective guided by one’s own intelligence, information, and deliberation; unreflective acquisition of knowledge is like lucking into some benefit in the dark (2009, p. 142).

Sosa is making two distinct claims here. The first is that reflective knowledge is more valuable than animal knowledge because reflecting on our beliefs makes them more reliable. The second is that reflective knowledge is more valuable than animal knowledge along a dimension other than truth. The thought is that reflective knowledge is a kind of success that is creditable to the knower in a way that animal knowledge is not.^11^ If either claim is right, then there is a difference in value between reflective and animal knowledge. We will look at each claim in turn.

The first claim should be understood as the claim that reflection *generally *increases reliability, not as the claim that reflection *always *increases reliability. As Sosa (1997) notes when discussing Moore’s (1959) justification for the reliability of perception, even the best sort of reflection isn’t going to promote reliability in a demon world where our first-order perceptual and other faculties are delivering us false beliefs *en masse. *Reflection on our first-order beliefs promotes reliability only when buttressing first-order competences that are themselves reliable as opposed to radically imperilled by deception. So: does reflection generally have a positive effect on the reliability of our beliefs (and if so, how)?

This matter is, as Kornblith rightly notes, subject to empirical refutation (or confirmation), and he offers some empirical evidence that he thinks shows that reflection does not generally increase reliability (see 2012, pp. 20–26). First, we are generally very bad at identifying the causes of our beliefs. Many of our belief-forming processes occur below the level of consciousness. Second, when we try to identify the relevant processes, we often make mistakes because our beliefs are often influenced by factors we mistakenly treat as irrelevant.^12^ For instance, Kornblith discusses evidence that suggests that our judgments about the reliability and trustworthiness of politicians are influenced by the colours of their campaign materials in ways that can’t be explained by political colour-coding (e.g. red $=$ left wing). Third, when we reflect on our beliefs, we confidently and entirely mistakenly end up rationalising them rather than uncovering these problems. As Kornblith puts it:

In a large class of cases, the process of reflection is an exercise in self-congratulation. It does nothing, however, in these important cases, to improve on the accuracy of our first-order beliefs (2012, p. 25).

Kornblith’s claim is that the empirical evidence suggests that reflection generally fails to increase the reliability of our reasoning.^13^ One might hope that a little reflection will reveal that one’s perceptions of politicians is influenced by irrelevant factors, but one would be wrong. It is fair to complain that Kornblith somewhat exaggerates the strength of the empirical evidence. Smithies (2016) cites several examples of cases where reflection can and does increase the reliability of our reasoning:

- Logical reasoning: Gagné and Smith Jr. (1962) found that verbalisation had a positive effect on problem-solving.
- Moral reasoning: Small et al. (2007) found that deliberation prevented individuals from donating more to ‘identifiable’ victims (but did not lead to them donating more to ‘statistical’ victims).
- Emotion regulation: Pennebaker and Chung (2011) found that reflecting on traumatic personal experiences has benefits for physical and emotional well-being.

While more could be said here, we would suggest that the jury is out on the epistemic benefits of reflection. The best that can be said is that reflection sometimes has epistemic benefits. While this doesn’t speak *against *Sosa, it hardly speaks in his favour. If the epistemic benefits of reflection are unclear, we can’t base our epistemology on the presumption of such benefits. So we now turn to Sosa’s second claim, which is that there is a special sort of value that attaches to reflective knowledge. Kornblith offers two objections here, which we call the *cognitive ideal argument *and the *conflicting aims argument*. The cognitive ideal argument proceeds as follows:

*Cognitive ideal argument*

1. If there is a special sort of epistemic value that attaches to reflective knowledge, then a maximally reliable and maximally unreflective agent would be epistemically suboptimal.
2. A maximally reliable and maximally unreflective agent is compatible with the cognitive ideal (viz., is not epistemically suboptimal).
3. Therefore, there is not a special sort of epistemic value that attaches to reflective knowledge.^14^

Premise (1) is surely true. But (2) is more problematic, for two reasons. First, one might think that a maximally reliable yet maximally unreflective individual must *ex hypothesi *not possess many or indeed any second-order truths that a maximally reliable and maximally reflective individual will have. Thus, unless second-order truths lack epistemic value entirely (which seems unlikely), it seems that the maximally reflective agent will be more epistemically optimal than the maximally unreflective agent. But then (2) must be false.

Second, it is at best contentious that epistemic optimality is secured by maximal reliability, even on a construal of maximal reliability where we suppose that one believes all truths. For one thing, as Jonathan Kvanvig (2003) argues, a cognitively ideal agent would possess maximal understanding, and it is contentious in epistemology whether understanding reduces to a sum total of knowledge, or whether it requires in addition a kind of grasping condition—or perhaps other kinds of cognitive features—that are not themselves secured simply through reliable believing, or for that matter through garden variety propositional knowledge.^15^ If these views are on the right lines, this spells serious trouble for (2).

Let’s now consider Kornblith’s *conflicting aims argument*:

*Conflicting aims argument*

1. Reflection often detracts rather than enhances reliability.
2. If (1), then aiming for reflective knowledge can put us at risk of failing to attain animal knowledge, which requires reliability.
3. Thus, aiming at reflective knowledge and aiming at animal knowledge are in normative conflict with one another: trying to achieve one may mean failing to achieve the other.^16^

Setting aside Smithies’ reasons for doubting (1), there is a deeper problem with this argument. It is not obvious why the conclusion is particularly troubling. Let’s grant Kornblith that the aims of achieving animal and reflective knowledge may sometimes conflict. But this is symptomatic of the more general fact (not distinctive of Sosa’s epistemology) that aiming for one genuine goal can put one at risk of failing to attain another. The most obvious instance of this phenomenon in epistemology is the twin injunctions that characterize the truth aim: to try to believe truths and to avoid believing falsehoods. Trying to achieve one aim puts one at risk of failing to achieve the other.^17^ Thus, the conflicting aims argument risks overgeneralizing.

Summarising, while Kornblith raises legitimate doubts about the empirical basis for Sosa’s claim that reflection promotes reliability, this hardly spells disaster for Sosa: more empirical work is needed. When it comes to the special value of reflective knowledge, Sosa is on more secure ground. The cognitive ideal argument at best begs the question, and the conflicting aims argument falsely presupposes that epistemic goals can never come into conflict. We tentatively conclude that Sosa can ground his distinction between animal and reflective knowledge in the distinctive value of reflective knowledge.

*Third Way: Anti-Sceptical Import*

If the reader is not convinced that the second way works, we offer a third and final way of grounding the reflective/animal knowledge distinction. Recall Kornblith’s argument that the first way of trying to ground the distinction would lead us to introduce a further category of knowledge: consultative knowledge. This is what Sosa says about the respective merits of reflection and consultation:

[W]hy put reflection above consultation? Partly, it seems to me, because the deliverances of consultation need assessing in the light of reflection in a way that is different from how reflection is to be assessed through consultation. In the end, reflection has properly a closer, more fully determinative influence on the beliefs we form, and the deliverances of consultation bear properly only through reflection’s sifting and balancing (Sosa (2004), p. 291).

While this point could be developed further, there is a stronger line of argument to be found in Sosa’s more recent work. One of Sosa’s central arguments for his bi-level virtue epistemology is that it has the resources to respond in a satisfying way to traditional sceptical challenges. In contrast, it is hard to see how a bi-level epistemology that distinguished between consultative and non-consultative knowledge would have similar resources. Sosa (2007, 2011) develops this thought in connection with *external world *scepticism; Sosa (2015, 2017) develops it in connection with *Pyrrhonian *scepticism. For simplicity we’ll focus on external world scepticism.^18^

Sosa is primarily concerned with *dreaming scepticism* rather than with scepticism based on evil demon or brain-in-a-vat scenarios because, in his view, the possibility that I might be dreaming is more of a threat to knowing anything about the external world than the possibility that I might be deceived by an evil demon or a brain in a vat. To see why, consider the two standard modal conditions on knowledge, safety and sensitivity:

safety: An agent S has a safe belief in a true contingent proposition p iff in most near-by possible worlds in which S believes p, p is true.

sensitivity: An agent S has a sensitive belief in a true contingent proposition p iff in the nearest possible worlds in which p is not true, S no longer believes p.

While the belief that I’m not a brain in a vat is insensitive (in a world in which I was a brain in a vat, I would still believe that I wasn’t), it is safe (in all nearby worlds in which I believe I am not a brain in a vat, I’m not a brain in a vat). Thus, many epistemologists—including, at one point, Sosa (see Sosa 1999)—have appealed to safety in order to ward off the threat of external world scepticism. But the belief that I’m not dreaming is plausibly neither sensitive nor safe. While worlds in which I am deceived by an evil demon or a brain in a vat are *modally distant*, worlds in which I am dreaming are plausibly *modally close*. They are, as Sosa puts it, “too close for comfort, given how naturally and often we dream” (2007, p. 99). Thus, one standard response to scepticism based on evil demons or brains in a vat is toothless when it comes to dreaming scepticism.^19^

Although Sosa doesn’t put it this way, one way of reading him is as arguing that his bi-level epistemology is necessary if we want to endorse four plausible but mutually inconsistent claims:

(i) The dream scenario is modally close.
(ii) We know (in some sense) a lot of things about the external world.
(iii) Barney (in some sense) doesn’t know that he is looking at a barn.
(iv) Knowledge (in some sense) requires safety.

Assume that there is no distinction between animal and reflective knowledge. There is only knowledge *simpliciter*. If [following (iv)] we want to put a safety condition on knowledge *simpliciter *then, while we can [following (iii)] explain why it is so plausible that Barney doesn’t know that he is looking at a barn despite having an apt belief (his belief is unsafe given the modal proximity of error), we are thereby forced to say that all subjects who have apt but unsafe beliefs lack knowledge. But, if [following (i)] the dream scenario is modally close, it follows that the vast majority of our beliefs are unsafe, and so we know very little about the external world.^20^

What happens if we reject this assumption? We can now say that Barney has animal knowledge, where animal knowledge is just apt belief, and a belief can be apt yet unsafe. Because one can have animal knowledge even if one’s belief is unsafe, dreaming scepticism is no threat to our animal knowledge. But we can also say that Barney lacks reflective knowledge, where reflective knowledge secures the safety of the relevant first-order belief.^21^ Thus, we can accept (i), and clarified versions of (ii)–(iv):

(ii*) We have lots of animal knowledge about the external world.^22^
(iii*) Barney lacks reflective knowledge (though has animal knowledge).
(iv*) Reflective knowledge ensures that the relevant first-order belief is safe.

If this is right, then one basis for Sosa’s reflective/animal knowledge distinction is that failing to make it leaves one in serious philosophical trouble. If one doesn’t distinguish between reflective/animal knowledge, one can’t endorse (i)–(iv); if one does, one can endorse (a version of) (i)–(iv).

In this respect the basis for Sosa’s distinction is similar to Ryle’s (1945, 1949) basis for distinguishing between knowing-how and knowing-that. Ryle’s thought was that, unless we distinguish between knowing-how and knowing-that, we will be faced with an intractable regress. Assume, for reductio, that knowing-how is a kind of knowing-that. On this assumption, if a pupil knows how to reason in accordance with modus ponens, this must be in virtue of knowing some fact(s) (e.g. about what must follow from what). But, Ryle insists, one may know such facts without knowing how to draw the required inferences, and familiar Carrollian considerations show that one can’t close this gap simply by adding further facts to the student’s body of knowledge. Ryle concluded that knowing-how can’t be a kind of knowing-that. Rather, knowing-how is a matter of possessing a sort of *ability*.^23^

Whether or not one agrees with Ryle about this, our point is just that Sosa’s reason for positing the reflective/animal knowledge distinction is similar. For Ryle, failing to distinguish between knowing-how and knowing-that leaves us unable to make sense of what is involved in reasoning in accordance with rules. For Sosa, failing to distinguish between reflective and animal knowledge forces us to choose between several individually plausible but jointly incompatible claims. In each case, the motivation is that the distinction is needed to solve a philosophical problem. Unless Kornblith can show that the consultative/non-consultative knowledge distinction does similar work, we should resist the invitation to lump Sosa’s distinction in with the others.

## Conclusions

We have argued—contra Kornblith—that Sosa’s distinction between reflective and animal knowledge satisfies the three adequacy conditions:

- It is part (indeed, a crucial part) of a plausible more general epistemological picture.
- It does not boil down to the familiar distinction between knowing (that p) and knowing that you know (that p).
- It is worth drawing insofar as it is based on the distinctive value of reflective knowledge, and the anti-sceptical import of a bi-level epistemology.

Of course, more work would need to be done to establish the overall plausibility of Sosa’s epistemological picture—we have only addressed a worry about Sosa’s treatment of fake barn cases. But, given the central importance of Sosa’s work to contemporary epistemology, we can conclude that we all have reason to take the idea that there is an important difference between human and animal knowledge seriously.

## Footnotes

1 This is essentially Sosa’s solution to the problem of epistemic circularity, which he attributes to Descartes (see Sosa 1997).

2 For discussion of Sosa’s ‘performance epistemology’ see the essays in Fernandez (2016).

3 Following Sosa’s own practice, we often won’t distinguish between reflective knowledge and knowing full well. His considered view is that knowing full well is the ‘best’ sort of reflective knowledge, and the central distinction is really between knowing full well and animal knowledge, not reflective knowledge and animal knowledge (see e.g. 2015, p. 75).

4 Put roughly, the exercise of one’s first-order epistemic competences is guided by the relevant meta-competences just in case one manifests the right kind of sensitivity to defeaters. One must, for instance, treat reasons to worry about the reliability of the relevant first-order competence to be reasons to worry about the beliefs one forms through exercising it.

5 For discussion (and criticism) of the KK principle in epistemology see Alston (1980), Greco (2014) and Williamson (2000). For discussion in formal epistemology see Hintikka (1962). For game theory see Lewis (1969).

6 Recent experimental work has cast doubt on the received view that knowledge should not be attributed to subjects in fake barn cases (see Blouw et al. (2017), Colaco et al. (2014), Turri et al. (2015) and Turri (2017)). While this raises important questions, we don’t think it helps Sosa in the present context.

7 This condensed formulation is taken from Pritchard (2012). For the classic presentations of the case, see Ginet (1975) and Goldman (1979).

8 Although Russell did not use this terminology, we might say that knowledge by acquaintance is an objectual knowledge relation, whereas knowledge by description is a propositional knowledge relation. For discussion of the epistemic features of objectual knowledge relations, see Bengson and Moffett (2011) and Benton (2017).

9 We set aside a fourth way, which appeals to the idea that reflective knowledge is implicated in epistemic agency (see Sosa 2009, p. 138). Kornblith has a simple response to this: there is no such thing as epistemic agency, only agency *simpliciter *(2012, pp. 31–34). We set this aside because the point of contention here is whether there is any need to posit an additional sort of agency, and this raises similar issues to those presently under discussion.

10 For some recent developments of this point, see Mi and Ryan (forthcoming).

11 To make sense of this, consider archery. Compare two archers who are equally good when it comes to the actual practice of taking a shot but differ in that one is very competent at choosing when to exercise their archery skills whereas the other is not competent. There seems to be a sense in which the successes of the first archer are more valuable than the successes of the second. Their successes are in some sense more creditable to them.

12 See Tversky and Kahneman (1973, 1975) for seminal discussions of the role of cognitive heuristics and biases in decision making. For discussion of the epistemological ramifications of the undetectability of such biases see Alfano (2012, 2014).

13 One potential line of reply, which we are setting aside for the present purposes, would be to dispute the *sense *of reflection that Kornblith is adverting to in this particular line of critique against Sosa. Kornblith is challenging the reliability of reflection, taken literally, and then by extension is calling into doubt the epistemic value of the reflective component of Sosa’s reflective knowledge. One problem, however, is that reflective knowledge, for Sosa, needn’t always involve *literal *reflection—viz., reflection that includes conscious awareness of one’s own mental states. After all, for Sosa, some cases of reflective knowledge will be ones where one aptly *takes for granted* that one’s first-order belief would be apt. This is a point Sosa emphasises in Sosa (2007) and elsewhere; it is important to his view because to insist that reflective knowledge requires actual conscious thought about one’s own mental states would overintellectualize a kind of knowledge that Sosa thinks is actually rather widespread. Nonetheless, *some *reflective knowledge, in Sosa’s sense, does plausibly involve literal reflection, and so we are for the sake of argument engaging with Kornblith’s objection directly.

14 See Kornblith (2012, pp. 26–34). This is our attempt to reconstruct his reasoning.

15 For relevant discussion see Grimm (2011), Hills (2009, 2016) and Riggs (2009).

16 See Kornblith (2012, pp. 26–34). Again, this is our attempt to reconstruct his reasoning.

17 This point was made forcefully by James (1896) and thereafter by (among others) Alston (1980), Carter et al. (2016), Fallis (2005) and Foley (1987).

18 Note that the argument that engagement with Pyrrhonian scepticism forces an animal/reflective knowledge distinction is importantly different from the argument from external world scepticism. For discussion see Sosa (2017, chaps 1, 3).

19 In places Sosa embraces a novel account of dreaming as imagining to deal with dreaming scepticism (e.g., 2007, chap. 1, 2017, chap. 2). However, he also appeals to his bi-level virtue epistemology to do the same job (e.g. 2007, chap. 2). It is unclear why Sosa pursues both solutions. Perhaps the thought is that we don’t want our epistemology to be hostage to the best account of dreaming.

20 That is, if we assume some form of the closure principle (roughly: if S knows that p, and that p entails q, then S is in a position to know that q).

21 We outlined why reflective knowledge secures first-order safety in §3. See Sosa (2017, pp. 76, 88) for further discussion of first- and second-order safety.

22
Sosa (2009) also argues that we can have reflective knowledge about the external world. For our purposes we don’t need to get into whether this argument is successful.

23 These points are made most forcefully in Ryle (1949, chap. 2). See Stanley (2011, chap. 1) for an overview.
